# Chilling requirement of Ribes cultivars

**DOI:** 10.3389/fpls.2014.00767

**Published:** 2015-01-07

**Authors:** Hamlyn G. Jones, Sandra L. Gordon, Rex M. Brennan

**Affiliations:** ^1^Plant Science Division, College of Life Science, University of Dundee at James Hutton InstituteDundee, UK; ^2^School of Plant Biology, University of Western AustraliaCrawley, WA, Australia; ^3^Soft Fruit Breeding Group, James Hutton InstituteDundee, UK

**Keywords:** blackcurrant, bud break, chill models, climate change, winter chill

## Abstract

It is usually thought that adequate winter chill is required for the full flowering of many temperate woody species. This paper investigates the sensitivity of blackcurrant bud burst and flowering to natural weather fluctuations in a temperate maritime climate, and compares a range of chill models that have been proposed for assessing the accumulation of winter chill. Bud break for four contrasting cultivars are compared in an exceptionally cold and in a mild winter in Eastern Scotland. The results confirm the importance of chilling at temperatures lower than 0°C and demonstrate that no single chilling function applies equally to all blackcurrant cultivars. There is a pressing need for further model development to take into account the relationship between chilling temperatures and warming temperatures occurring both during and after the chill accumulation period.

## INTRODUCTION

It has been widely reported that adequate winter chilling is required for release from dormancy, regular bud break and flowering of many temperate woody species to occur in the subsequent year. This chilling requirement is also a protective measure to prevent woody species from growing in adverse winter conditions, and affects the geographical distribution of woody species (Sherman and Beckman, 2003). Additionally, the levels of winter chilling that are required are variable between and also within species, such as *Prunus* (Valentini et al., 2004), *Vaccinium* (Song et al., 2013), and *Ribes*. Blackcurrant (*Ribes nigrum* L.) has been identified as a temperate woody fruit species that has a particularly high chilling requirement, and as a consequence recent warm winters in the UK have lowered yields and fruit quality, partly as a result of uneven ripening (Jones et al., 2013). Blackcurrant has been grown commercially in the UK for over 100 years (Brennan, 2008), and the juice processing industry has expanded during the latter part of the 20th century, including in northern areas, due in part to the introduction of new frost-tolerant cultivars (Brennan, 1991). However, increasing concern about the damaging effects of reduced winter chilling in blackcurrant has led to an increased focus by breeders on the production of new cultivars with more resilience to changing climatic conditions.

Major questions remain regarding what constitutes a chilling temperature with suggestions that this varies with species and even cultivar. Such information is critical if we are to account for chill in order to predict the potential effects of climate change, or to optimize selection of varieties for any particular climatic region or climate change scenario. It is convenient to treat dormancy as a sequential process, with a requirement for chilling to satisfy the endodormancy requirement and then a subsequent warming requirement to enable bud break (satisfying ecodormancy; Cesaraccio et al., 2004, 2006). Heat requirement (HR) was considered by Richardson et al. (1974) and Citadin et al. (2001) as a major factor determining the subsequent time of flowering in *Prunus* spp., although it remains unknown whether heat accumulation for flowering and bud break begins before or after the release of endodormancy.

A wide range of chilling functions or chilling ‘models’ have been proposed to account for the accumulation of chill in different species. The most commonly used functions have been (i) the total number of hours below 7.2°C, (ii) the total number of hours between 0 and 7.2°C, and (iii) the ‘Utah’ units which accumulate chill using a weighting function that emphasizes temperatures between 0 and 7°C. Lantin (1973, 1977) recognized that such units do not well reflect the chilling response of blackcurrants which appear to be more sensitive to lower temperatures and developed an exponential chilling function based on that proposed initially by Bidabe (1967). Harrington et al. (2010) proposed a model based on a minimum number of chilling units (termed critical chilling requirement), below which budburst does not occur, and an optimum chilling requirement, after which additional chilling has no effect on timing of budburst. Clearly, between these two points, there can be various combinations of chilling and heat units that can lead to budburst.

It is feasible in principle to estimate chill requirements from a statistical analysis of bud burst in the field in relation to prevailing weather conditions, by taking data from many years and locations, but this approach tends to be rather unreliable as it is difficult to get a representative range of weather conditions. The approach can be improved by transferring cut shoots to an ‘enabling’ temperature environment so as to eliminate complications in the field arising from the warming requirement. More rigorous data can be obtained by imposing the chill to shoots or to whole plants in controlled temperature chambers at a wide range of chilling temperatures (Sunley et al., 2006). Unfortunately this latter approach does not readily lend itself to evaluation of responses to chill in naturally fluctuating environments, and these fluctuating temperature conditions have become increasingly normal in winters in northern area (Henry, 2008; Luterbacher et al., 2009). Erez and Lavee (1971) demonstrated chilling negation by higher temperatures in peach, and subsequently Erez et al. (1979a,b) showed that the level of negation was highly temperature-dependent, with 8 h of high temperatures negating *ca*. 16 h of chilling temperatures in a daily cycle. Also, the stage of chilling at which the high temperatures were applied was significant, as has been confirmed in apple by Young (1992).

In this paper we compare the performance of a small number of diverse blackcurrant cultivars in two very contrasting years, characterized by widely disparate levels of chilling at key stages throughout the winter. From this, we have compared the effectiveness of different chill models in explaining the correlations between winter temperature fluctuations and budburst.

## MATERIALS AND METHODS

### PLANT MATERIAL

Plant material was grown in open field plots at the James Hutton Institute at Invergowrie, Dundee, Scotland. Full experimental details for the 2007–2008 experiment have been provided elsewhere (Jones et al., 2013), but in brief, in contrast to the present field experiment, dormant shoots of the cultivar ‘Ben Gairn’ were harvested from the field in October 2007 and transferred to controlled environment chambers maintained at –5, 0, 5, or 10°C for varying periods, at the end of which they were transferred to a glasshouse at 20°C to allow bud development. Final bud burst was recorded after 45 days warming in the glasshouse.

For the experiments in 2012–2013 and 2013–2014, 4-year old blackcurrant bushes of the Scottish-bred cultivars ‘Ben Gairn,’ ‘Ben Dorain,’ and ‘Ben Tirran,’ and the New Zealand-bred cultivar ‘Murchison’ were again grown in open field plots. These cultivars represent different cropping seasons and flowering times: ‘Ben Gairn’ and ‘Murchison’ are usually considered as low-chill requiring types (Brennan, 2008), with an early flowering and harvest date, while ‘Ben Dorain’ and ‘Ben Tirran’ require higher levels of winter chill and flower and ripen considerably later.

Six hardwood cuttings (*ca*. 15 cm length) were taken from three bushes of each cultivar at weekly intervals from October to field bud break in March during 2 years (2012/2013 and 2013/2014). In the first year, the six cuttings were not assigned to individual bushes, whereas in 2013/2014 two cuttings were taken from each bush. The cuttings were brought into a glasshouse at 20°C, where they were kept in individual boiling tubes containing water under natural daylight conditions. Bud break was monitored and scored using the average of the top 12 buds on a shoot. Bud break was defined as the point at which green leaf material was visible on the buds. Overall the genotype differences in final budburst achieved following any harvest date were significant (*P* < 0.05) on most dates over the season (excepting the first two sample dates and a few others in each year) as analyzed in Minitab16 (Minitab Ltd., Coventry, UK) using ANOVAs for each day using arc-sin transformed data (one-way ANOVAs for 2012–2013, and nested ANOVAs for 2013–2014. In addition, data on the mean date of first attainment of each of the four stages (Bud break, Leaves visible, first Open flower and Full flowering) were recorded in each year for plants maintained in the field.

### METEOROLOGICAL DATA AND CHILL CALCULATION

Hourly temperature records were obtained from a site in Auchterhouse, Dundee (*ca*. 4 km from the blackcurrant plots). From this data, amounts of winter chill were calculated as follows:

• 0–7.2°C units (denoted by ‘0–7.2’) – total hours summed from 1 October in the relevant year where the mean temperature was between 0 and 7.2°C
• < 7.2°C units (denoted by ‘<7.2’) – total hours summed from 1 October in the relevant year where the mean temperature was less than 7.2°C
• exponential units (denoted by ‘exp’) – where ‘exp’ is the hourly sum from 1 October of the function ‘exp’ = 0.6702*[exp(–0.148*T_a_)], where *T*_a_ is the hourly mean temperature. This function was derived by curve fitting to the temperature response of chilling in 20 similar blackcurrant genotypes in a previous study (Jones et al., 2013)

We have not included in this study detailed analysis of any of the other chill units that have been proposed, such as the ‘Utah’ units, because earlier work has clearly shown their lack of relevance for blackcurrant (Sunley et al., 2006), or the more complex dynamic models that also take account of warming in the field (Erez and Fishman, 1998; Luedeling et al., 2013), because the latter ideally require calibration over several years for local conditions.

## RESULTS

### CHILL ACCUMULATION

**FIGURE 1:**
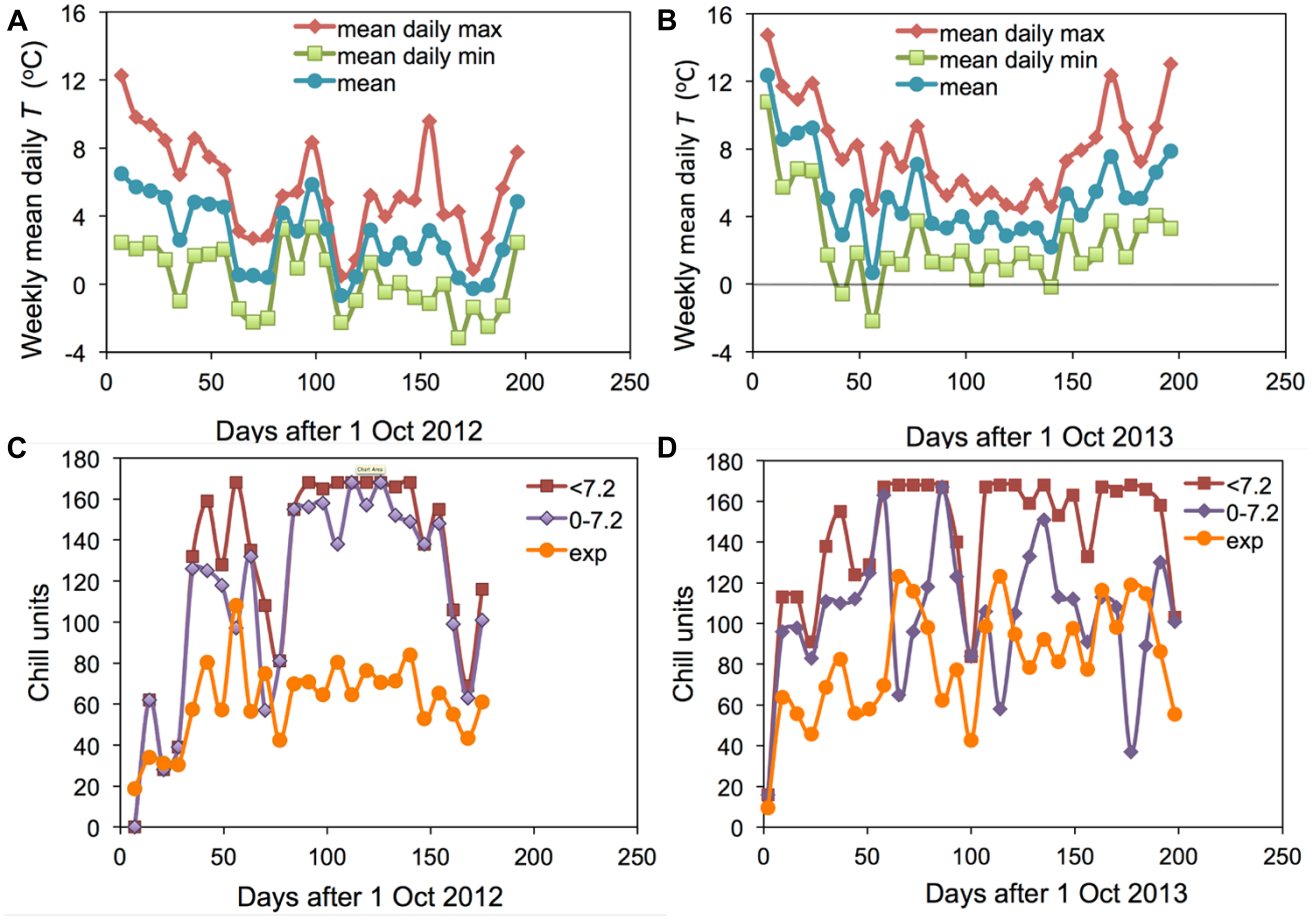
**Weekly means of the daily mean, daily maximum and daily minimum temperatures for Auchterhouse for **(A)** 2012–2013 and **(B)** 2013–2014**. Comparable data on the weekly accumulation of the hourly values of three separate chill units (‘<7.2,’ ‘0–7.2’ and ‘exp’) are shown in **(C)** 2012–2013 and **(D)** 2013–2014.

**FIGURE 2:**
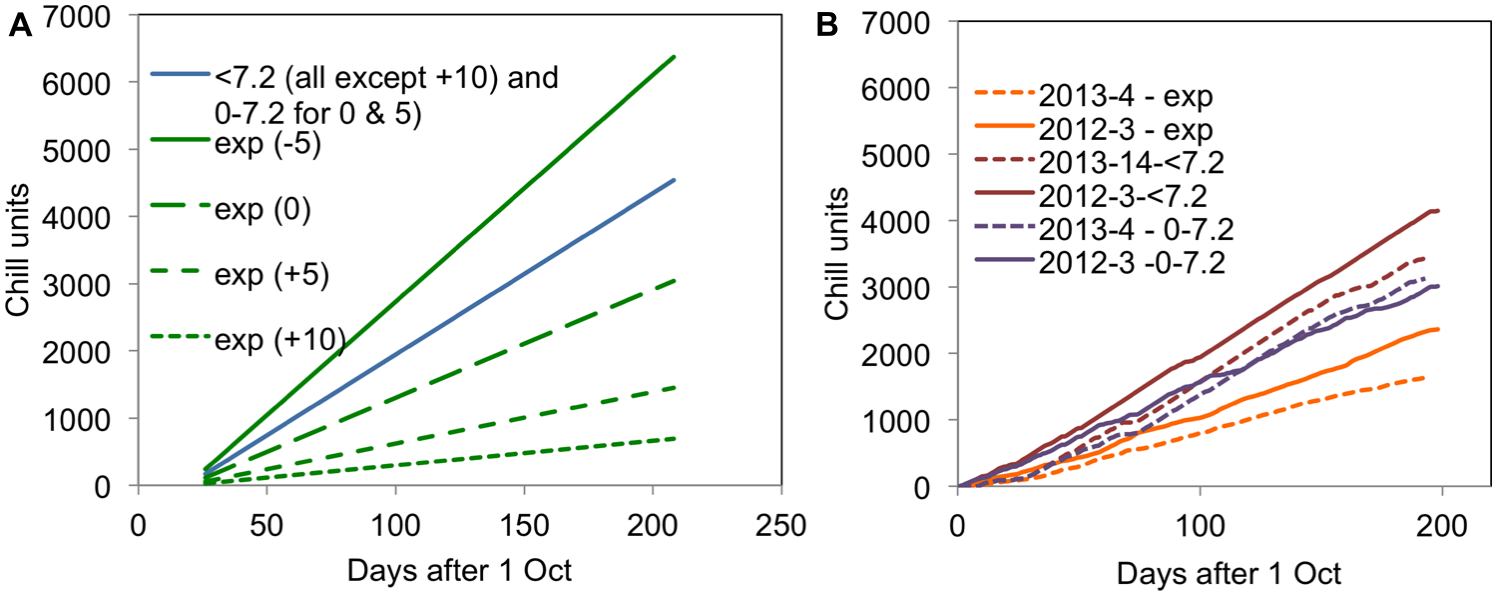
**Accumulation of different chill units for **(A)** the 2007–2008 experiment in the four CE temperature treatments and **(B)** in 2012–2013 and 2013–2014 for the field experiments**. All units are accumulated from 1 October in the relevant year.

The two winter seasons investigated in this study had contrasting conditions: 2012–2013 was an exceptionally cold winter and 2013–2014 was the warmest winter on record in Angus. The weekly means of daily maximum, minimum and mean temperatures at Auchterhouse are shown for the 2 years in **Figures 1A,B**. The corresponding mean temperatures over the 27 weeks following 1 October were 2.8 and 5.2°C, respectively. However, the accumulation of chill calculated as hours < 7.2°C was rather similar in the 2 years (except for a short period in early October) leading to an end of season value only 17% lower in the warmer 2013–2014 (**Figure 2B**; compare **Figures 1C,D**). On the other hand, the warmer year actually accumulated 4.5% more hours between 0 and 7.2°C than did the cooler year (when a large proportion of days were below the critical value of 0°C). It is only the accumulation of exponential units (or Lantin units which emphasize the lower temperatures) where the colder winter accumulated substantially more ‘chill’ (43% more). A similar effect is found if only hours below 0°C are accumulated (data not shown).

It is particularly interesting to note that the accumulation of <7.2 and 0–7.2 units was almost the same in the warmer year (2013–2014) but very different in the cold year of (2012–2013). This result suggests that it would not be possible to distinguish the <7.2 and 0–7.2 chill units statistically in a warm year such as 2013–2014. The field accumulation of chill in the two field experiment years is compared with the experimental controlled environment treatments applied in the 2007–2008 experiment (Jones et al., 2013) in **Figure 2**. This figure shows that the exponential chill accumulation for the 0°C treatment was slightly more rapid than the actual field accumulation in the colder year.

### CONTROLLED ENVIRONMENT RESULTS FOR 2007–2008

**FIGURE 3:**
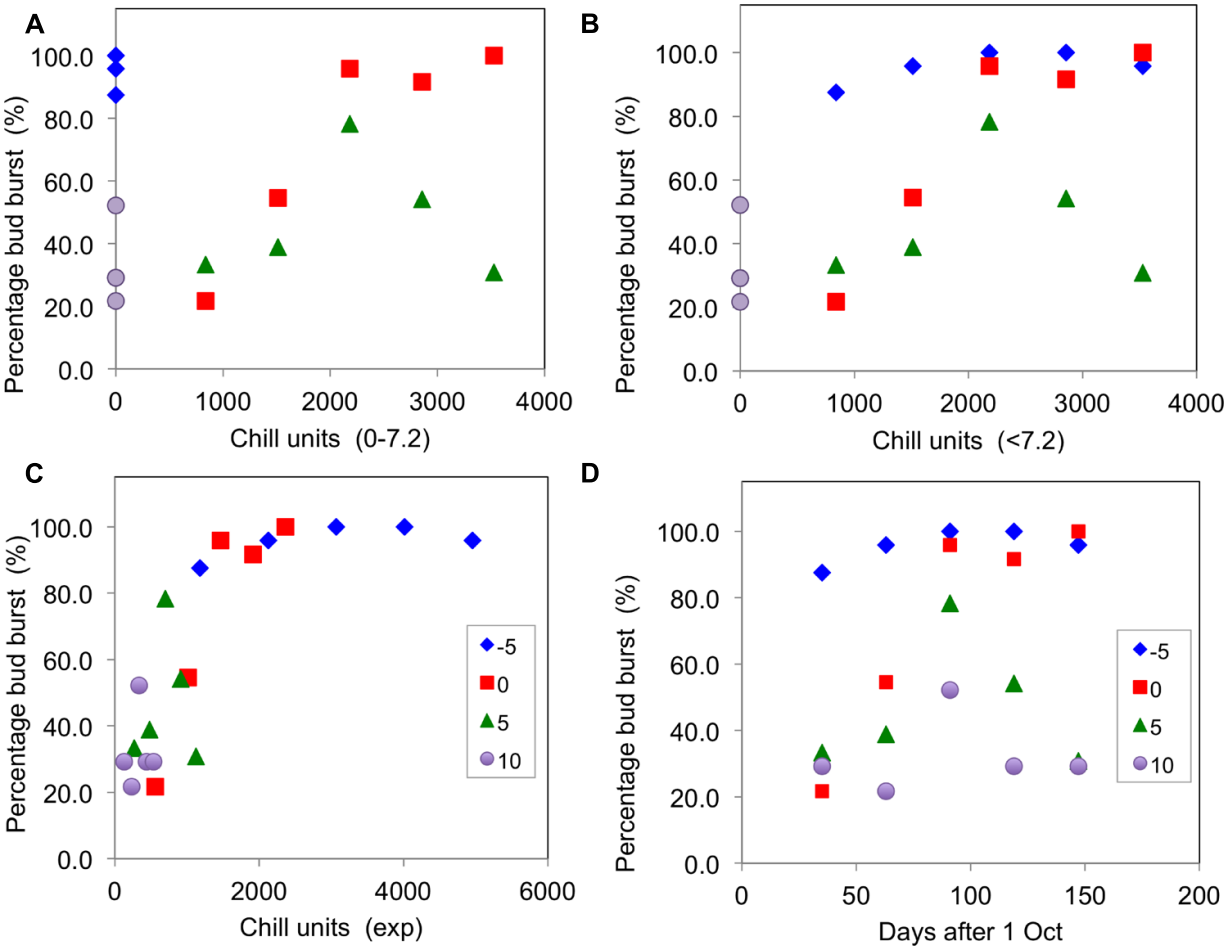
**Percentage buds open (recorded after 45 days warming) plotted against chill units accumulated for ‘Ben Gairn’ in the 2007–2008 experiment in the four CE temperature treatments: **(A)** plotted against 0–7.2°C units, **(B)** plotted against <7.2°C units, **(C)** plotted against exponential units accumulated and **(D)** plotted against time (days after 1 October)**.

As a simple test of the main chill units that have been proposed, **Figure 3** presents plots of the percentage of buds open for the blackcurrant cultivar ‘Ben Gairn’ (recorded after a period of 45 days warming – adequate to complete any warming requirement) against chill accumulation for the controlled environment experiment of 2007–2008. This figure shows that budburst was particularly poorly related to hours of chill accumulation for 0–7.2°C units (**Figure 3A**) as full bud burst occurred without any chill accumulation for the -5°C treatment and some bud burst even occurred for the 10°C treatment. There were also clear differences in effectiveness for the 0 and 5°C treatments, indicating that some weighting for temperature is probably required. Similarly the <7.2°C units were also not well related to effect (**Figure 3B**), with differing effectiveness of the -5, 0, and 5°C treatments although they all ostensibly accumulated the same amount of chill. On the other hand, there was a generally good association between budburst and chill accumulation for all treatments with the exponential units (**Figure 3C**). It is noteworthy that plotting budburst against days of chilling (**Figure 3D**) showed decreasing effectiveness from -5°C, through 0°C, and 5°C to 10°C. It is also worth noting that an optimum budburst actually occurred with the two warmer treatments partway through the season, with the amount of budburst decreasing with longer treatments at 5 and 10°C.

### FIELD DATA ON BUD BURST – COMPARISON OF COLD AND WARM WINTERS

The median dates of reaching four different stages of bud opening for the four blackcurrant cultivars in the field are summarized in **Table 1** for the cold winter of 2012–2013 and the warm winter of 2013–2014. Note that actual budburst in the field was substantially later for all cultivars in the exceptionally cold winter of 2012–2013 than in the mild winter of 2013–2014. Although rigorous data are not available, observation suggests that the spread of dates of bud burst in the field for any one cultivar was substantially greater in the warmer year (2013–2014), also probably as a result of the smaller amount of chill received that year. It is notable, however, that the range between the dates for the different cultivars was not substantially different in the 2 years (except for the earliest bud break stage).

**Table 1:** Dates of first visible attainment of four bud development stages for four blackcurrant cultivars in the field at Invergowrie in the 2012–2013 and 2013–2014.

	Bud break	Leaves visible	First open flower	Full flower
**Winter 2012–2013**
‘Ben Gairn’	15-March	11-April	29-April	16-May
‘Murchison’	05-April	15-April	02-May	13-May
‘Ben Dorain’	05-April	15-April	09-May	23-May
‘Ben Tirran’	19-April	25-April	20-May	31-May
Mean/range	*3-April/35 days*	*16-April/14 days*	*7-May/21 days*	*21-May/18 days*
**Winter 2013–2014**
‘Ben Gairn’	10-March	24-March	14-April	28-April
‘Murchison’	17-March	31-March	14-April	28-April
‘Ben Dorain’	17-March	07-April	28-April	05-May
‘Ben Tirran’	31-March	14-April	05-May	19-May
Mean/range	*19-March/21 days*	*3-April/21 days*	*22-April/19 days*	*5-May/21 days*

*Also shown are the mean dates for each stage and the range (d) between the cultivars.*

**FIGURE 4:**
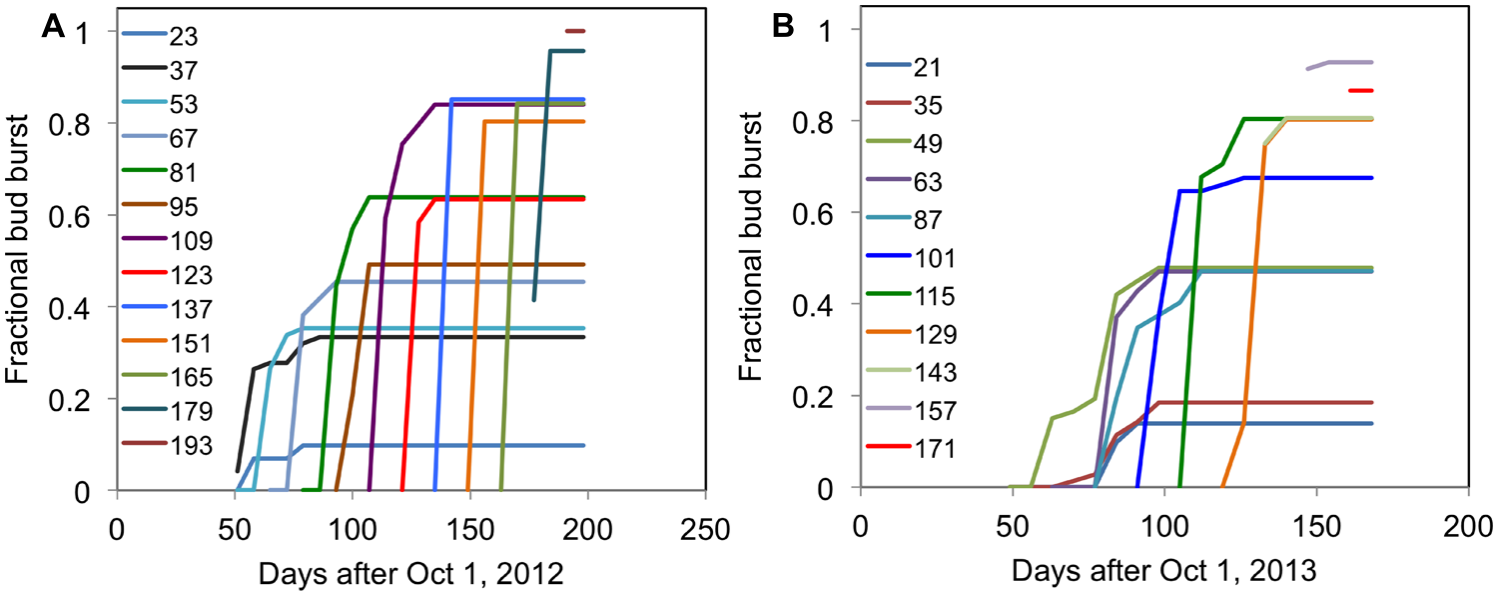
**Each line shows the time course of fractional bud break for cuttings of blackcurrant (‘Ben Gairn’) harvested from the field at fortnightly intervals (indicated for each line as days after 1 October) and transferred to 20°C **(A)** for 2012–2013 and **(B)** for 2013–2014**.

The typical time course of the fraction of buds broken (where buds open are expressed a fraction of total buds) for ‘Ben Gairn’ in two contrasting years when harvested from the field at different dates and brought into an enabling environment at 20°C is shown in **Figure 4**. For any harvest date the fraction of buds breaking saturates at a fixed amount irrespective of how long they are subsequently retained under enabling conditions at 20°C. In subsequent graphs, therefore, we only present the final fraction of bud break achieved after a period at 20°C.

**FIGURE 5:**
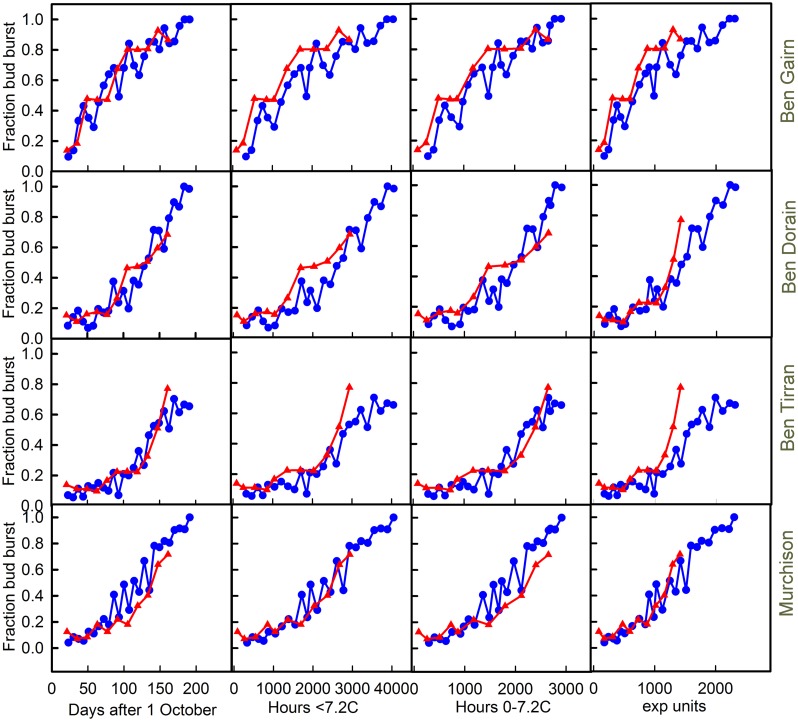
**Plots of bud break for each of the cultivars [‘Ben Gairn’ (row 1), ‘Ben Dorain’ (row 2), ‘Ben Tirran’ (row 3) and ‘Murchison’ (row 4)] plotted against either days after 1 October (column 1), accumulation of ‘<7.2’ chill (column 2), ‘0–7.2’ chill (column 3) and ‘exp’ chill units (column 4)**. Blue lines with circles refer to 2012–2013, while red lines and triangles refer to the 2013–2014 data.

The effects of harvest at different dates on final bud break in 2012–2013 and 2013–2014 are shown for each of the four cultivars in **Figure 5**. In each case the final fraction of buds bursting is plotted against each of the three chill units and also against harvest date. Note that this method of data presentation removes the effect of the warming requirement that confounds observations of the final date of bud burst in the field.

Any substantial differences in the relationship between budburst and chill accumulation in the two contrasting years provides good evidence for a failure of the chilling model being used as a predictor. The chill units that gave the best agreement between years varied with cultivar. In spite of the very different winter temperatures in the 2 years it is interesting to note that plotting bud burst against time is almost as good as plotting against any chill unit accumulation. Indeed for Ben Gairn, the best agreement between years is for a plot against date, suggesting that actual chill is not critical for this cultivar. For ‘Ben Tirran’ the 0–7.2 unit gave the best correspondence between years, while the exponential chill accumulation was best for ‘Murchison.’ No unit worked well for ‘Ben Dorain’ whose bud burst potential rose consistently earlier in the warm year. On the other hand, it is clear that for ‘Ben Tirran’ and ‘Murchison’ bud burst potential rose earlier in the cold winter (2012–2013) than in the warm winter.

A notable feature of the results shown in **Figure 5**, which include a period of controlled warming, is the contrast with the results of field records shown in **Table 1**. For example, although all cultivars flowered later in the field in the colder year (2012–2013), ‘Ben Tirran’ and ‘Murchison’ showed earlier bud break in in 2012–2013 than in 2013–2014 when the confounding effect of the warming phase is eliminated (**Figure 5**).

## DISCUSSION

The work conducted in this study covered two very contrasting winters in Northern Britain, from one of the coldest winters in recent years in 2012/2013 to undoubtedly one of the warmest in 2013/2014. Such variability of winter conditions is increasingly characteristic of the northern European maritime climate. Interestingly, although there were large differences in the amount of time below freezing, the total number of hours below 7.2°C was fairly similar in both winters. The temperatures in the colder winter were closely approximated by the controlled environment treatment used in the 2007–2008 experiment (Jones et al., 2013) where plants were maintained at 0°C.

Conventionally it is assumed that a single chill unit can be used to discriminate effectively between the chill requirements of different cultivars with different cultivars being separated on the basis of the number of units of chill required for effective flowering. On the assumption that chilling is a prerequisite for budburst and adequate flowering, a convenient method for comparison of the utility of different chill unit calculations is to plot the time course of budburst against chill accumulation: for an appropriate chill unit the lines for any 2 years should be coincident. Comparison of such plots for the two extreme winters indicated that the most suitable model to fit the data for the four cultivars in this study differed with both cultivar and year, highlighting the fact that none of the existing models are well-suited to variable field conditions. These results in themselves indicate that adherence to models based on simple temperature thresholds is unlikely to relate to field observations across a range of germplasm in different years.

The need for further development and refinement of models for winter chilling in woody plant species is linked to the increasing temperatures predicted through climate change as summarized by the International Panel on Climate Change (Solomon et al., 2007; Stocker et al., 2013). Indeed, in many areas including California and the UK, the most obvious trends in overall global warming are seen in the daily minimum temperatures during the winter months (Sunley et al., 2006; LaDochy et al., 2007). The overall impacts on the dormancy and development of woody perennial species are inevitably complex and require modeling that can take into account aspects such as timing of warming during the dormant period as well as overall temperature means. Luedeling et al. (2009), comparing different models of winter chill, advocated the use of a dynamic model based on the work of Erez et al. (1990) and Erez and Fishman (1998), and questioned the biological significance of models based on chilling hours alone. Unfortunately effective parameterisation of such models for any species requires data for many years. The fact that some genotypes were relatively little separated by natural chilling in the two experimental years (even when eliminating confounding effects due to the ecodormancy phase by warming in controlled environments) supports the hypothesis that chilling is not the only environmental factor affecting budburst as has been found by previous workers for other woody species (Heide, 1974, 1993), with daylength variation being a prime candidate as another relevant factor.

As climate change may lead to a delay in the onset of chill accumulation, this may also affect the time at which perennial plants become receptive to heat during spring (Luedeling et al., 2013). This is partly borne out by the observations of budburst and subsequent development observed in this study: **Table 1** shows that the dates of actual budburst observed in the field averaged 2 weeks earlier for all cultivars in the warm winter of 2013–2014 than in the exceptionally cold winter of 2012–2013. We hypothesize that at least part of the delay in the cold winter may not relate to chill satisfaction, but to fact that the warming phase was delayed by the very late spring in that year. This conclusion is supported by the different results observed when controlled warming was provided. Although flowering was advanced in all four cultivars after the warmer winter of 2013/2014, the genotypic differences in flowering date in the field were largely maintained over the 2 years. The differing responses of the four cultivars used may in part be explained by their relative genetic backgrounds. In particular, the New Zealand cultivar ‘Murchison’ is derived from a low-chill environment, and requires significantly less winter chilling overall than a high-chill Scottish cultivar such as ‘Ben Dorain.’ However, the differences in time of bud break between the cultivars was considerably less following the warmer winter than the colder one, although by flowering this trend was reversed. Despite the genetic differences, the work by Harrington et al. (2010) in pine suggests that the biochemical factors involved in the sensing of chilling are likely to be similar across many species, but the development of suitable species-specific models is still required to accurately quantify some aspects.

Phenotyping of the plant material has to date focused mainly on the chilling temperatures between October and March, with relatively less attention on the effects of warming temperatures occurring both during endodormancy and also as the plants move toward bud break. Such information will be needed to improve the accuracy and applicability of models of chilling and bud break, and to allow wider phenotyping to be undertaken. A particularly useful approach will involve the screening of material grown at different locations (Cesaraccio et al., 2001, 2004). Whether future research should attempt to fit final flowering or the full seasonal dynamics into the model depends on the eventual use of the model, as the latter may be considered of less relevance to plant breeders and to climate scientists, only being of specific relevance to physiologists interested in the mechanism of endodormancy.

Interestingly, it was found by Castède et al. (2014) in *Prunus* that while the correlation between chilling requirement and flowering date is significant and constant, the correlation with HRs showed a much higher variability between years. There would therefore seem to be a strong case for the provision of more detailed and multi-site phenotyping with particular focus on the impact of warmer temperatures at key points within the chilling accumulation phase, since the work of Erez et al. (1979a) demonstrated the significance of the timing of warming temperatures.

Work to understand the underlying genetics linked to chilling and dormancy release has been reported in *Prunus* spp. (Sanchez-Perez et al., 2012; Castède et al., 2014). Similar work is in progress in blackcurrant, using both individual cultivars and also mapping populations and genomic resources developed in *Ribes* (Hedley et al., 2010). In blackcurrant, the availability of significant diversity of response to chilling and warming temperatures during the winter gives some optimism that the application of contemporary genomics and molecular breeding techniques can accelerate the production of new cultivars with resilience to future climate scenarios. However, such work is dependent on accurate and relevant phenotyping, and the future development of more robust and dynamic models for the analysis of responses to winter chill and dormancy release is an essential part of this process. The work both to develop robust chilling models and also to understand the genetic control of chilling accumulation and bud break is not only of considerable significance to the future sustainability of blackcurrant production in Europe, but it also has generic relevance across a range of woody species, both crop and landscape, in the context of a changing climate.

## Conflict of Interest Statement

The authors declare that the research was conducted in the absence of any commercial or financial relationships that could be construed as a potential conflict of interest.
